# African Swine Fever Laboratory Diagnosis—Lessons Learned from Recent Animal Trials

**DOI:** 10.3390/pathogens10020177

**Published:** 2021-02-06

**Authors:** Jutta Pikalo, Paul Deutschmann, Melina Fischer, Hanna Roszyk, Martin Beer, Sandra Blome

**Affiliations:** Institute of Diagnostic Virology, Friedrich-Loeffler-Institut, 17493 Greifswald-Insel Riems, Germany; Jutta.Pikalo@fli.de (J.P.); Paul.Deutschmann@fli.de (P.D.); Melina.Fischer@fli.de (M.F.); Hanna.Roszyk@fli.de (H.R.); martin.beer@fli.de (M.B.)

**Keywords:** African swine fever virus, laboratory diagnosis, genome detection, antibody detection, sample matrix, blood swabs

## Abstract

African swine fever virus (ASFV) causes a hemorrhagic disease in pigs with high socio-economic consequences. To lower the impact of disease incursions, early detection is crucial. In the context of experimental animal trials, we evaluated diagnostic workflows for a high sample throughput in active surveillance, alternative sample matrices for passive surveillance, and lateral flow devices (LFD) for rapid testing. We could demonstrate that EDTA blood is significantly better suited for early ASFV detection than serum. Tissues recommended by the respective diagnostic manuals were in general comparable in their performance, with spleen samples giving best results. Superficial lymph nodes, ear punches, and different blood swabs were also evaluated as potential alternatives. In summary, all matrices yielded positive results at the peak of clinical signs and could be fit for purpose in passive surveillance. However, weaknesses were discovered for some matrices when it comes to the early phase of infection or recovery. The antigen LFD showed variable results with best performance in the clinical phase. The antibody LFD was quite comparable with ELISA systems. Concluding, alternative approaches are feasible but have to be embedded in control strategies selecting test methods and sample materials following a “fit-for-purpose” approach.

## 1. Introduction

African swine fever virus (ASFV), a large, enveloped, double-stranded DNA virus, which belongs to the genus *Asfivirus* within the *Asfarviridae* family, causes an often fatal hemorrhagic disease in domestic pigs and wild boar with high socio-economic consequences worldwide [1]. Over the past decade, the disease has spread to several European and Asian countries and is still moving further, putting pig industry and the connected value chain at stake [2]. 

For early detection of ASF and timely implementation of control measures, targeted sampling of sick and dead animals, i.e., passive surveillance, is of utmost importance [3,4]. This is particularly crucial because of the fact that the disease is associated with high lethality, but also moderate or even low morbidity and mortality [5]. The latter is linked to contagiosity that can be moderate in wild boar populations or larger domestic pig farms in the absence of parenteral transmission routes by competent vectors [6,7,8]. The animals to be sampled in passive surveillance are obviously sick or have died, so it can be assumed that a significant viral load is present in several organs and tissues [6]. Direct detection methods have priority to detect the disease. With this in mind, and considering that ASFV is highly stable even in decaying carcasses [9,10], pragmatic approaches for sample collection, suitable sample matrices, and reliable testing can be discussed that could facilitate compliance and thus efficient early warning. Along these lines, several approaches have been assessed in the recent past. Specifically, the applicability of different dry blood swabs [11,12], dried filter papers and FTA cards [13,14,15], fecal samples [16], oral, nasal and rectal swabs [17], meat-juice [18], and different rope-based options [19,20] has been assessed. Further matrices such as intraocular fluid, superficial lymph nodes (e.g., inguinal lymph nodes), ear punches following the example of BVDV diagnosis [21], and the like have been discussed. 

Apart from passive surveillance, high-throughput active surveillance and monitoring are still needed in affected countries with intensive pig industry and/or high density of wild boar. To this means, random sampling of live animals or the wild boar hunting bag is applied, and healthy animals with a low probability of infection are the large majority. Under these circumstances, low expected virus prevalence is linked to low viral loads, and antibody detection should be included [22]. Here, the choice of the most reliable and resource-saving sample matrices can also be crucial. 

In the context of a series of animal experiments with strains of different ASFV genotypes and defined endpoints within the acute phase of ASFV infection, i.e., 4 to 10 days post infection (DPI), we took the opportunity to compare and evaluate diagnostic workflows for both active and passive surveillance. Our focus was primarily on qPCR detection of ASFV genomes. In particular, we investigated the possible limitations of serum as sample matrix for monitoring purposes, compared different organs and tissues of wild boar and domestic pigs for their viral loads, and evaluated alternative sample matrices that could be used in the context of passive surveillance in domestic pigs and wild boar.

Finally, we investigated the performance characteristics of “point-of-care" or “pen-side” diagnostics for both ASFV antigen and antibody detection.

## 2. Results

### 2.1. Samples Taken from Domestic Pigs and Wild Boar Are Comparable

**Figure 1:**
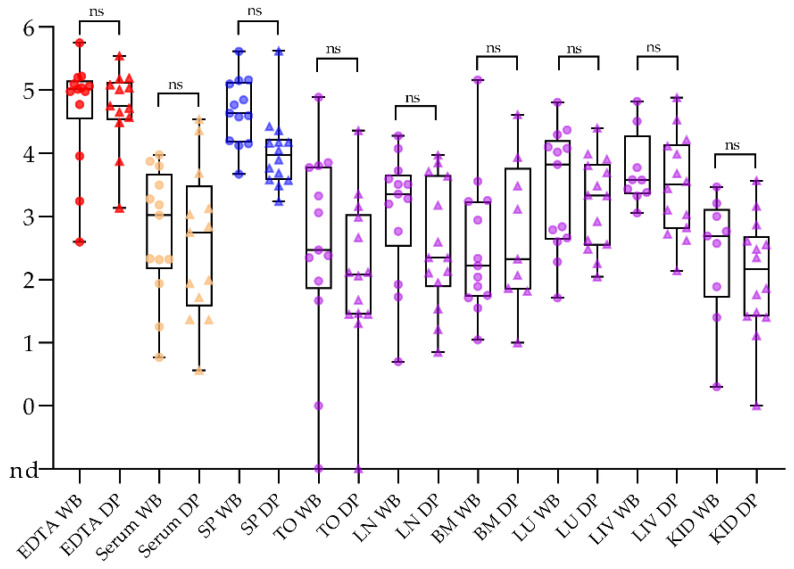
Comparison of sample matrices taken from wild boar (WB; dots) and domestic pigs (DP; triangles). The qPCR results are depicted as log_10_ genome copy numbers per run. Abbreviations: nd = not detected; SP = spleen, TO = tonsil, LN = lymph node, BM = bone marrow, LU = lung, LIV = liver, KID = kidney, ns = not significant in pairwise comparison.

Our sample set (see Supplementary Table S1) comprised samples from domestic pigs (n = 37) and European wild boar (n = 16). Therefore, it had to be clarified whether the samples were comparable and thus evaluable together. Taking the post infection data set of all wild boar and the directly corresponding domestic pigs (n = 13 each), none of the tested sample matrices showed significant differences (see Figure 1 and Supplementary Figure S1). All downstream analyses were therefore performed with both wild boar and domestic pigs in one combined data set. 

### 2.2. Serum May Reach Its Limits for Active Surveillance

In the attempt to limit the sampling effort to one matrix with low inhibitory effects in qPCR, high potential for automation, and general suitability for all direct and indirect diagnostic tests, serum was evaluated in detail. One aspect was the comparison with EDTA blood as a standard matrix that is known to contain high viral loads.

At all sampling days, positive and valid qPCR results were obtained for all EDTA blood samples and for all but one serum sample taken from inoculated domestic pigs and wild boar. Control animals remained negative. Thus, EDTA would ensure 100% sensitivity in the given test system, serum reaches only 98%. No problems arose with the internal control system applied (heterologous control). Over the entire comparison, considerably higher genome loads were found in EDTA blood samples at all times and in all animals. The difference was most obvious in the early phase of the ASFV infection (4 DPI) where serum samples contained genome copy numbers as low as 4 or 6 copies per run. In this experimental phase, five out of six animals yielded copy numbers below 100. In the phase of obvious clinical signs, serum contained also higher genome loads but these loads were still much lower than in EDTA blood. The difference was higher again at 10 DPI. The single negative serum originated from an animal that had shown a subclinical disease course upon infection with a genotype IV ASFV strain. All individual results (experimental background and genome copy numbers per run) are depicted in Supplementary Table S1. 

**Figure 2:**
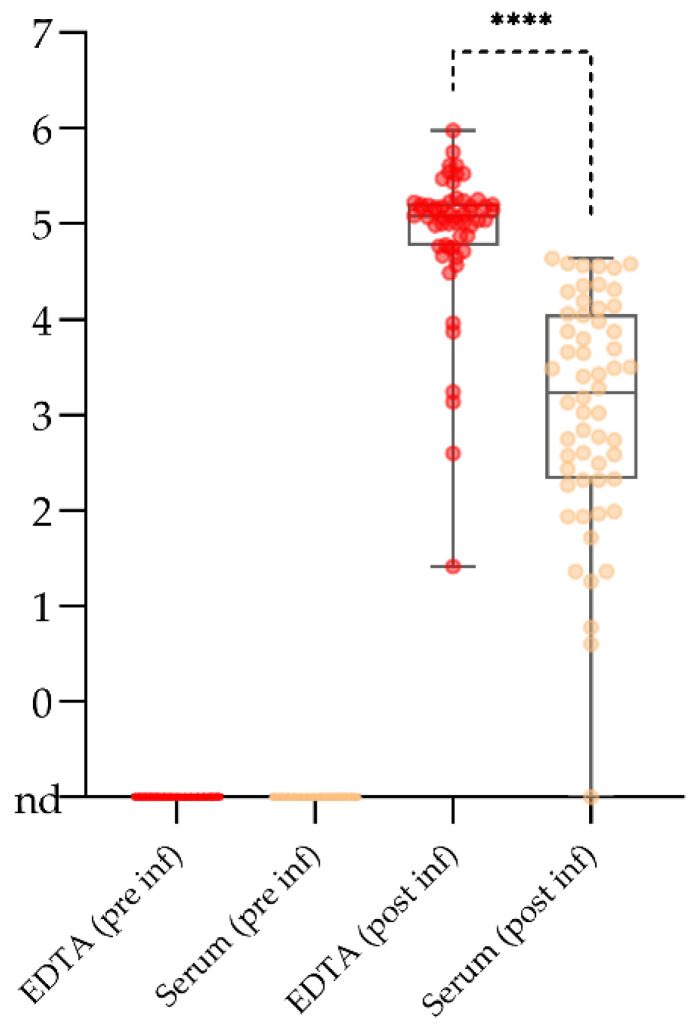
Overall comparison of log_10_ genome copy numbers in EDTA blood and serum (prior to infection = pre inf; post infection = post inf). The boundaries of the boxes indicate the 25th and 75th percentiles, the line within the box marks the median. Whisker boundaries indicate minimum and maximum values. A paired t-test was performed to test the significance with a resulting **** *p*-value of < 0.0001 for samples taken post infection. nd = not detected.

Comparing the overall genome loads at all time points (53 pairs), EDTA blood showed significantly higher (*p* value < 0.0001) values post infection (see Figure 2). Especially in the early phase, serum was close to the limit of detection and the mean genome loads in EDTA blood were roughly 200 times higher (see Supplementary Table S1). As no false positive reactions occurred, performance with negative samples was not significantly different (see Figure 2, EDTA and Serum pre inf). 

### 2.3. No Surprise in the Comparison of Routine Post Mortem Sample Matrices 

**Figure 3:**
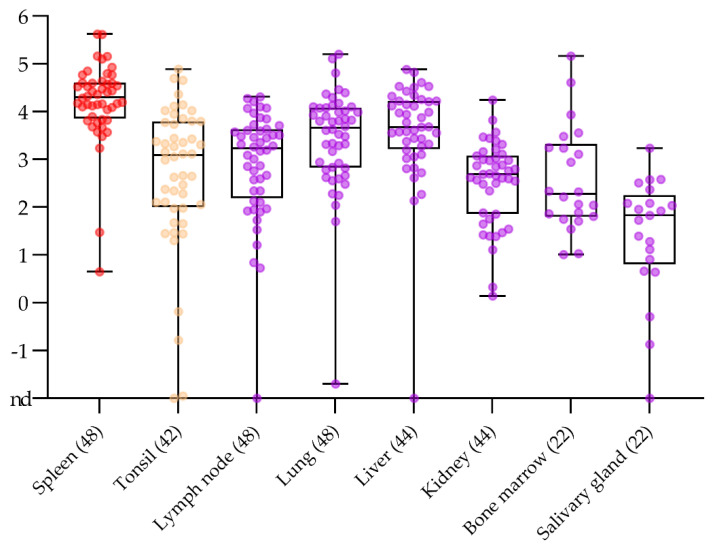
Comparison of log_10_ genome copy numbers per run in different organs over the entire data set. The numbers in brackets indicate the number of animals included for the respective matrix. All samples are individually depicted together with the box plot. The boundaries of the boxes indicate the 25th and 75th percentiles, the line within the box marks the median. Whisker boundaries indicate minimum and maximum values. Lymph node: mandibular/sub-mandibular lymph node; salivary gland: parotis. nd = not detected.

Standard organs for passive and active surveillance, i.e., tonsils, spleen, mandibular lymph nodes, bone marrow, lung, liver, and salivary glands, were analysed and compared for the presence and the amount of ASFV genome (see Figure 3). 

Over the whole data set, spleen samples gave consistently positive results with rather high genome loads that reached a maximum of 4.2 × 10^5^ genome copies per run (see Figure 3 and Supplementary Table S1). The genome loads in spleen were significantly higher than in tonsils (*p* value 0.0016), lymph nodes (*p* value 0.0007), lung (*p* value 0.0116), liver (*p* value 0.0100), and kidneys (*p* value 0.0007). Not considering the large difference in sample numbers for bone marrow and salivary gland versus spleen (22 vs. 48), pairwise comparison showed no significant difference between bone marrow and spleen (*p* value 0.0507) but a significant difference between salivary gland and spleen (*p* value 0.0148). Individual false negative results were observed with samples taken from tonsils, lymph nodes, salivary glands, liver, and kidney. Three out of five false negative results were obtained from one animal. The same animal gave a false negative result using serum (see above). No false positive results were obtained from control animals (see Figure S1). Considering sensitivity (disregarding quantitative differences), spleen, bone marrow, and lung reached 100%. A sensitivity of roughly 98% was reached using tonsils, lymph nodes, kidney, and liver. Resulting from the smaller sample size, sensitivity of salivary gland samples was 95.5%.

### 2.4. Alternative Sample Matrices for Passive Surveillance in Domestic Pigs and Wild Boar

#### 2.4.1. Sampling Fallen Domestic Animals without Opening Body Cavities in the Stable 

Superficial lymph nodes, ear punches, and ocular fluids were investigated as sample matrices for fallen domestic animals upon the request of (German) veterinary authorities and practitioners in pig-dense areas. 

**Figure 4:**
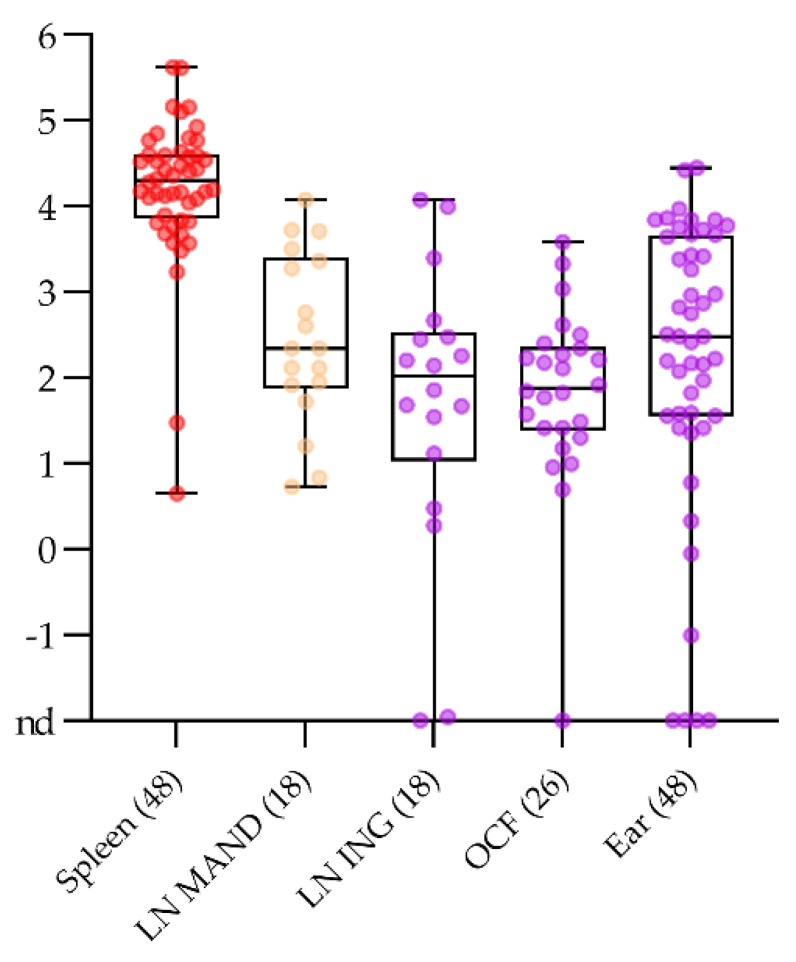
Comparison of log_10_ genome copy numbers per run in spleen, inguinal lymph nodes (LN ING), and mandibular lymph nodes (LN MAND), ocular fluids (OCF), and ear punches (ear). All samples are individually depicted together with the box plot. The boundaries of the boxes indicate the 25th and 75th percentiles, the line within the box marks the median. Whisker boundaries indicate minimum and maximum values.

Among the lymph nodes that are easiest to access without opening the abdominal cavity, or the need to cut deep into the carcass, are inguinal lymph nodes. Their suitability for ASF diagnosis was assessed in comparison with the best choice sample spleen and the mandibular lymph node that could also be taken without opening any body cavities. For this comparison, the data set was restricted to the comparative study with ASFV strain “Estonia 2014” where different lymph nodes had been separated (n = 18 samples per matrix). In summary, all samples gave positive results in qPCR. However, the variability was highest and the genome load lowest for the inguinal lymph node. Values far below one copy (at the detection limit) to roughly 10^4^ genome copies per run were observed. For the mandibular lymph node, a rather low variability was observed with a mean copy number of 1.8 × 10^6^ per run. Comparative data are depicted in Figure 4.

With regard to ear punch samples, all 53 animals were included in the comparison, 48 animals post infection and 5 controls. Forty-four out of 48 samples (92%) taken post infection were found positive for viral genome in low to moderate amounts (trace amounts to 3 × 10^4^ with a mean of 3.5 × 10^3^). No false positive reactions occurred in the controls. 

Ocular fluids (aqueous humour) was sampled from 26 animals. The sampling was difficult and the final sample matrix was rather undefined material from the interior of the eye than aqueous liquid. However, all but one animal (96%) gave a positive signal with rather low genome loads (mean 3.8 × 10^2^). The negative animal was again the one already described for other sample matrices. 

A comparison of the above-mentioned alternative sample matrices with spleen samples is depicted in Figure 4. Spleen showed significantly higher genome loads than any of the tested alternative matrices. A significant difference was also observed between the mandibular lymph nodes and the ocular fluid (*p* value 0.0330). No significant differences were seen among the other alternatives.

#### 2.4.2. Blood Swabs Can Still Be Optimized 

**Figure 5:**
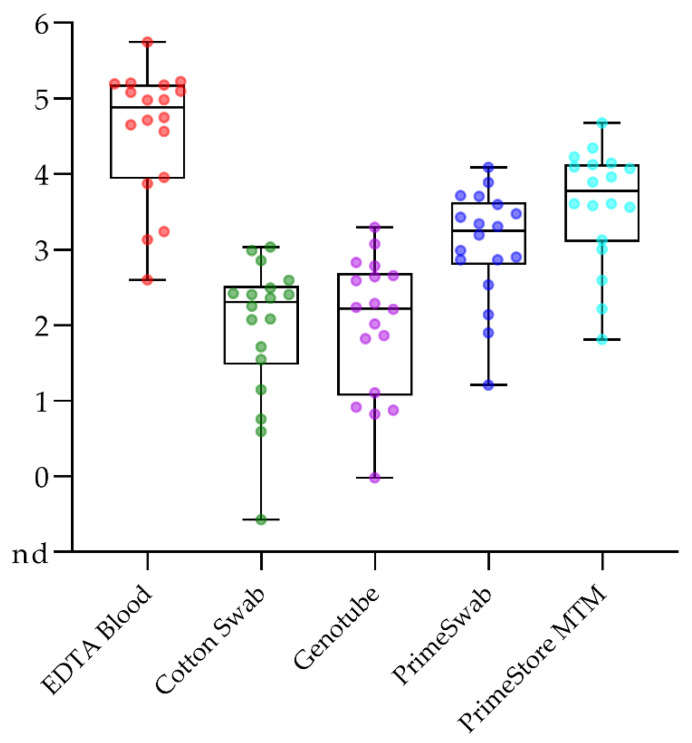
Comparison of genome copy numbers in different swab options and EDTA blood as comparator. Samples were taken from wild boar (WB) and domestic pigs (DP) over the entire time of the experiment. All samples are individually depicted together with the box plot. The boundaries of the boxes indicate the 25th and 75th percentiles, the line within the box marks the median. Whisker boundaries indicate minimum and maximum values. PrimeSwab indicates the swab itself, PrimeStore MTM the accompanying transport buffer.

Continuing previous studies [11,12,23], we compared and evaluated different blood swab options. Along with the previously tested plain COPAN cotton swabs (cotton swab) and GenoTube Livestock Swabs (Genotubes), PrimeSwabs and the inactivating PrimeStore MTM transport buffer were included in the assessment. Comparison was done with EDTA blood as standard matrix, and among the different swab and swab buffer options. For this study part, matched samples were available from the comparative trial with ASFV “Estonia 2014”. Taken the entire data set of domestic pig and wild boar samples from this trial, all tested matrices of infected animals gave positive results. However, EDTA blood contained significantly higher viral genome loads (*p*-value < 0.01). Comparing the different swab options, viral genome loads varied significantly. Plain cotton swabs and Genotubes gave weakest results with several samples that contained only trace amounts or less than 10^2^ genome copies per run. Both PrimeSwabs and PrimeStore MTM buffer performed significantly better. Comparing PrimeSwab and PrimeStore MTM buffer directly, the MTM buffer performed best and significantly better than any other swab option, including the PrimeSwab (*p*-values ranging from 0.02 to 0.003). An overview is presented in Figure 5. No significant differences were observed again between domestic pigs and wild boar (see Supplementary Figure S2). All control animals were tested negative with all swab options (see Supplementary Table S2).

The direct comparison of the alternative matrices (superficial lymph nodes, ear punches, ocular fluid, and swab options) underlines the good performance of blood swabs and the inactivating transport buffer (see Supplementary Figure S3). 

### 2.5. “Point-of-Care” Tests for Ressource-Limited Settings and as a Tool for Epidemiological Investigations

The presented study included the use of commercial lateral flow devices (LFD) for the detection of ASFV antigen or antibodies in the comparative trial using ASFV “Estonia 2014”. To assess both sensitivity and specificity, all samples were incorporated, irrespective of the sampling day (0 to 10) and the anticipated outcome. 

#### 2.5.1. Lateral Flow Devices for ASFV Antigen Detection Have Limitations but Yield Positive Results in the Clinical Phase

The antigen LFD was assessed with EDTA blood and serum as sample matrix, and the results were compared with the outcome of standard qPCRs. At 4 DPI, only one domestic pig showed a questionable LFD result using serum and a positive LFD result using EDTA blood. The reactive animal was also the one with the highest genome loads (691 copies per run in serum, 1.5 × 10^5^ copies in EDTA blood). In the phase of overt disease, at 7 DPI, almost all samples gave positive LFD results using either serum or EDTA blood of domestic pigs or wild boar. One domestic animal showed a negative LFD result when using EDTA blood (but a positive result with serum). The negative result was not linked to a significantly lower content of viral genome although it was in the EDTA blood taken that day (5.6 × 10^4^ copies per run). At 10 DPI, all serum samples were found positive with weaker results that corresponded in the majority of cases with lower genome copy numbers in qPCR. When taking EDTA blood as a matrix, two domestic pigs were found negative. These animals were the ones with rather the lowest genome copy numbers. However, these copy numbers were much higher than for positive sera. A summary of visual results and their interpretation is presented in Supplementary Figure S4 and Table S2.

The attempt to optimize the outcome for EDTA blood samples through freeze-thawing or dilution in distilled water did not yield better results. 

#### 2.5.2. ASFV Antibody Lateral Flow Devices Show Promising Results with Samples Taken from Recovering Animals

The antibody LFDs were also used with both EDTA blood and serum. The results were compared to three commercial antibody enzyme-linked immunosorbent assays (ELISA) that are routinely used in the laboratory. Moreover, indirect immunoperoxidase tests were used for final confirmation.

All samples taken at 4 DPI and 7 DPI were found negative in all assays applied for antibody detection, including the indirect immunoperoxidase test. At 10 DPI, all three domestic pigs showed positive LFD results when using serum as sample matrix (see Supplementary Figure S5). These results corresponded to positive results in all ELISA assays (see Supplementary Table S3). Two of these animals were also positive when applying EDTA blood. The later results corresponded to positive or questionable results in all ELISAs. The remaining domestic pig showed a negative result with EDTA blood. However, the same animal showed positive or questionable results in the ELISA tests. The questionable results were found in an indirect ELISA format. The wild boar showed a more heterogeneous reactivity. Testing serum, a negative, a weak positive, and a questionable result were obtained. There was also heterogeneity in ELISA results (see Supplementary Table S3) with highest positive rates in competitive formats. Using EDTA blood, two weak positive and one negative result were obtained. Also with this sample matrix, higher heterogeneity was observed in the ELISA, and negative results were obtained in the indirect format (see Supplementary Table S2). The overall results of antibody detection corresponded to the observation that the domestic pigs were already recovering at 10 DPI while the wild boar were still showing signs of disease. The indirect immunoperoxidase test was positive for all animals sampled at day 10 confirming their status as positive. 

## 3. Discussion

Because of its impact on animal health and pig industry, ASF is considered as one of the most important viral diseases of domestic pigs and wild boar. In the absence of commercial vaccines or treatment options, timely detection and implementation of control measures is of utmost importance [22]. The clinical manifestation of ASFV infection is usually most severe in domestic pigs and Eurasian wild boar [24]. However, most signs are highly unspecific and therefore, laboratory diagnosis is mandatory to confirm any clinical suspicion [25]. 

Over the past decade, the disease has gone pandemic and has reached not only the world’s largest pig producer [26] but also several other countries with considerable pig production in both Asia and Europe. An additional layer of complexity is added through the involvement of wildlife with wild boar as a reservoir in several European countries [27]. Surveillance activities in pig-dense areas can mean tremendous sample numbers and optimization of diagnostic workflows is of utmost importance to direct human and financial resources in a senseful manner, especially in times of other pandemic diseases of high significance that also demand diagnostic resources. In this context, limitation to one single sample matrix for *intra vitam* laboratory diagnosis has been discussed and one of the favored matrices under Central European conditions would be serum. In Germany and other Central European countries, collection of native blood from hunted wild boar has its roots in classical swine fever surveillance and was also applied for domestic pigs. Serum is a rather robust matrix that can be put on automated extraction and ELISA systems, and is suitable for all direct and indirect swine fever tests (both African and classical swine fever). Apart from being suitable for all antibody detection methods, inhibitory effects in qPCR are lower in serum than in anticoagulated blood [28]. Quality can be an issue when sampling is performed by hunters, but this also applies to other sample matrices. Against this background, we tested the suitability in the early, clinical, and later phase of ASFV infection in comparison with EDTA blood. Given the fact that ASFV has usually hemadsorbing capacities and is attached to erythrocytes [29,30], it is not surprising that there is a significantly higher load of viral genome in EDTA bloods samples. Yet, our experience from previous trials showed that serum was comparable in overall diagnostic sensitivity as long as clinically diseased animals were sampled (unpublished data accompanying the study reported by Gabriel et al. [31]). Here, animals in the early, pre-clinical phase, animals showing almost no obvious signs of disease, and animals that were showing first signs of recovery were included. With these samples, serum got to its limits and considering our results, we could not recommend using serum for the screening of apparently healthy animals (e.g. in restriction zones). Especially when planning to use any pooling of samples, false negative results have to be expected. As a consequence, the German official method collection for notifiable diseases was amended regarding the sample matrix for ASF diagnosis in animals without obvious clinical signs or lesions. Taking EDTA blood as the standard matrix may require some optimization regarding PCR inhibition [28] and use of certain extraction methods in larger settings. For passive surveillance, serum is probably fit for purpose. In only one of our samples taken at 7 DPI or later, results got close to the detection limit of the PCR. This one animal was also negative in several other matrices and was only picked up reliably in spleen and blood. It should be also kept in mind that the moderate virulence of some of the virus strains used in our experiments could have influenced assay sensitivity in the early phase. Comparing trials with ASFV “Armenia08” and “Estonia 2014”, there is roughly a ten-fold lower genome load in the early phase. An advantage of serum is definitively the suitability for virus isolation. Toxic effects and contamination are seen much less frequently with serum than with organ samples or blood.

Regarding tissue samples, all matrices recommended by the diagnostic manuals of the World Organization for Animal Health (OIE) [32] or the EU [33] gave reliable results with highest viral genome loads in spleen, lung, and liver, as expected for a virus that replicates in myelomonocytic cells including circulating monocytes and tissue macrophages [34,35]. However, endothelial cells [35], megakaryocytes [36], and parenchymal cells like hepatocytes [35] among others, also proved to be permissive for ASFV which is also reflected by the outcome of the tissue comparison. Quite surprisingly, tonsil samples were less homogeneous, especially in the early phase of the infection. This is contradictory as the tonsil is one of the primary replication sites [37]. It cannot be excluded that the texture of the sample, i.e., the coarse nature, and our decision to test in a diagnostic manner without biological replicates led to poor homogenization and release of less viral nucleic acids for extraction. In this respect, spleen, lung, and liver were easiest to work with. The salivary gland was taken into the set of samples under the assumption that shedding through saliva would be accompanied by the presence of viral genome in the gland tissue. Considering our results of high variability and rather low genome loads, the salivary gland will remain a matrix for scientific studies targeting shedding of ASFV. 

The sample matrices described above are routine for veterinary practitioners or pathologists. However, if passive surveillance is the most important tool for early detection of ASF [3,4,7], alternative samples may be better suited, especially for carcasses. In the European Union, the Commission implementing decisions [38] direct the sampling toward fallen animals that occur in a farm. In this context, samples that could be taken without the need to open the body cavities of the carcass would be beneficial in terms of environmental contamination. For this reason, we investigated inguinal lymph nodes, ocular fluid, and ear punches, especially for the domestic pig setting. The inguinal lymph node gave rather reliable results as can be expected from this tissue type. However, variability was high, and in the early phase, genome loads close to the limit of detection were observed. An explanation could be that the virus was not yet distributed to peripheral sites. However, this would not be in line with the antigen detection in popliteal lymph nodes in the same study [39] and thus, sampling error, i.e., inclusion of fatty or connective tissues of the inguinal region, cannot be completely excluded. Ocular fluid was difficult to sample and genome loads were low. In our hands, this matrix was not practicable. Ear punches of clinically diseased animals were positive for viral genome which is in line with recent findings that also show the skin yielded positive results when testing wild boar carcasses [40] or experimentally infected animals [17]. However, the low level of viral genome and the quite difficult handling does not make this matrix an alternative candidate for routine settings. 

Over the last years, our group has validated blood swabs as an alternative matrix for passive surveillance, especially in wild boar [11,12,23]. Only recently, the approach was also put to field practice when ASF entered Germany, and it performed well [40]. As optimization is always possible, and new development have been put on the market, we include a new type of swab and transport buffer into our comparison. The PrimeSwab and the accompanying PrimeStore MTM lived to our expectations and performed best in the comparison. This system has been evaluated using both bacterial and viral pathogens, including SARS-CoV-2 and is intriguing because of the safe inactivation of pathogens and preservation of nucleic acids [41,42,43,44]. Whether it is worth using this system or its sequels (PrimeStore HCP) instead of simple swab systems, remains the choice of users based on risk assessment, integration into strategies, and financial resources. 

In summary, our results add to the data body that alternative sample matrices could be considered. Among the published options that were not further followed up in the presented study are oral fluids, faecal samples, and swabs as well as meat juices. While shedding will depend on the virulence of the isolate [45], most secretions and excretions will be positive for ASFV genomes in the clinical phase [17]. When it comes to antibody detection, oral fluids were shown to work with a slight delay in detection [46] and faeces worked in principle but with high limitations [47]. Meat juice has proven to be a good matrix for the sero-surveillance of bacterial, protozoal, and viral diseases. With certain limitations, this also applies to the detection of ASFV- and ASFV-specific antibodies [18,48]. 

Our last focus was on the lateral flow assays that could aid diagnosis in resource-limited areas or help with rapid results during epidemiological investigations. In a nutshell, performance of antibody lateral flow devices was again rather comparable to ELISAs and the promising results that are published for similar assays could be confirmed [49]. Nevertheless, antibody detection might not be the most important part for ASFV point-of-care approaches. In the latter context, antigen detection would be the key focus. Our results showed that clinically diseased animals have a fair chance of being positive for viral antigen in the LFD. However, sensitivity is low and a negative result would need confirmation if signs or epidemiological settings would suggest ASF. Overall, further testing under field conditions is needed to conclude on the acceptability of ASFV antigen LFDs under different conditions. 

In summary, routine matrices performed best, but some alternative sample matrices deserve attention and could be part of well-designed surveillance strategies. So far, lateral flow devices for antigen detection require careful use and further investigations. 

## 4. Materials and Methods 

### 4.1. Experimental Design

The study comprised defined sample materials from domestic pigs and wild boar that were collected during an animal experiment where the animals had been oro-nasally inoculated with 2 × 10^5^ hemadsorbing units 50% (HAU) of ASFV “Estonia 2014”. This genotype II strain originates from Estonian wild boar [50] and shows moderate virulence [51] with a tendency of more severe disease courses in wild boar. For this reason, the strain was chosen for a comparative study on clinical outcome and pathology that was recently published by Sehl et al. [39]. Another aim of the study was generation of well-defined sample matrices for diagnostic test validation. The samples presented here were taken from nine domestic pigs and nine wild boar that were sequentially euthanized at 4, 7, and 10 DPI. Two domestic pigs and three wild boar were included as negative controls and were euthanized at 0 DPI. The sample set comprised EDTA anticoagulated blood, plasma, serum, spleen, tonsil, mandibular and inguinal lymph nodes, bone marrow, lung, liver, salivary gland, and the ear. The blood samples were additionally used to generate swab samples using different devices, the ear was used to create punches with commercial ear-tag tongs (see below). 

To complete the sample set for this study, further samples from different animal experiments were analyzed (see Supplementary Table S1): (1) samples from four wild boar and five domestic pigs that had been oro-nasally inoculated with 2 × 10^5^ HAU of ASFV “Belgium 2018/1”. This ASFV strain belongs also to the p72 genotype II showing high virulence in both species. In this study, samples were collected at the humane endpoint before euthanasia (between 8 DPI and 10 DPI). The sample set comprised EDTA anticoagulated blood, plasma, serum, spleen, tonsil, lymph nodes, bone marrow (wild boar only), lung, liver (domestic pigs only), salivary gland (wild boar only), kidney (domestic pigs only), intraocular fluid (domestic pigs only) and the ear. (2) Samples taken from domestic pigs intramuscularly inoculated with different African ASFV isolates that were kindly provided by Dr. Christopher Netherton (The Pirbright Institute, Pirbright, UK), i.e., five animals inoculated with 10 HAU of genotype IV strain “RSA W1/99” (South Africa [52]) and euthanized 8 DPI, five animals inoculated with 10 HAU of genotype XII strain “MFUE 6/1” (Zambia [52]) and euthanized at 7 DPI, five animals inoculated with 10 HAU of genotype XIX strain “CHZT 90/1” (Zimbabwe) and euthanized 7 DPI, three animals inoculated with 1000 HAU of genotype XI strain “KAB 6/2” (Zambia [52]) and sampled 8 DPI, and three animals inoculated with 1000 HAU of genotype XIII strain “SUM 14/11” (Zambia [52]) and sampled 8 DPI. The sample set for these additional animals comprised EDTA anticoagulated blood, serum, spleen, tonsil, lymph nodes, lung, liver, kidney, intraocular fluid (aqueous humour) and the ear. The clinical score which defined human endpoints was determined using the protocol described in Pietschmann et al. 2015 [53] with slight modifications.

All domestic pigs were bought from commercial pig farms and were clinically healthy upon arrival. The wild boar originated from different game parks and were purchased in healthy condition. All animals were tested negative for ASFV- and ASFV-specific antibodies prior to enrolment in the studies. 

The initial animal experiments for strain characterization and reference material collection were approved by the competent authority (LALLF, Rostock, Germany) under reference number 7221.3-2-011/19.

### 4.2. Processing of Samples and Preparation of Swabs

From the first animal trial where the animals (domestic pigs and wild boar) had been inoculated with ASFV “Estonia 2014”, samples were taken for both pathogen and antibody detection including preparation of swabs and use with lateral flow devices. Serum was obtained from native blood samples through centrifugation for 20 min at 2031× *g* at room temperature and was stored together with aliquoted EDTA blood samples at −80 °C until further usage. To obtain plasma for confirmatory testing, separate EDTA blood aliquots were centrifuged as described above.

Tissue samples from all animal trials were collected and aliquoted during necropsy and stored at −80°C prior to further use. For the ear punch samples, ears were punched with the FlexoPlus R ear tagging system (Caisley, Bocholt, Germany). In preparation of nucleic acid extraction, all tissue samples were homogenized for 3 min at 30 Hz in 1 mL phosphate-buffered saline (PBS) with a metal bead using a TissueLyser II (Qiagen^®^GmbH, Hilden, Germany).

Three types of commercial swabs were used to generate blood swabs: (1) plain cotton swabs (Rayon, COPAN, Brescia, Italy), (2) GenoTube Livestock Swabs (Thermo Fisher Scientific, Waltham, MA, USA), and (3) PrimeSwabs (Longhorn, Vaccines and Diagnostics, San Antonio, TX, USA). GenoTube Livestock swabs are equipped with a collection tube that rapidly dries the sample to avoid degradation of nucleic acids. The PrimeSwab is a flocked swab and compatible with PrimeStore Molecular Transport Medium (MTM). This MTM (Longhorn, Vaccines and Diagnostics), is a buffer based on guanidine thiocyanate that provides for virus inactivation and nucleic acid stabilization upon transport and storage.

All swab types were directly dipped into vials of whole blood upon or shortly after sampling. The COPAN cotton swabs (Rayon, COPAN, Brescia, Italy) and the GenoTube Livestock (Thermo Fisher Scientific, Waltham, MA, USA) devices were placed back into their receptacles, and the PrimeSwabs (Longhorn, Vaccines and Diagnostics, San Antonio, TX, USA) were placed into PrimeStore MTM (Longhorn, Vaccines and Diagnostics, San Antonio, TX, USA) tubes. All samples were then stored at room temperature for five days prior to further processing to mimic transfer of samples from the field to the laboratory. After storage, small pieces (2.5 mm in diameter) were excised from all blood swabs with sterile scissors and processed like tissue samples. In addition, the PrimeStore MTM buffers, in which the PrimeSwabs had been submerged, were used for nucleic acid extraction following the protocol for fluid samples.

### 4.3. Detection of Viral DNA

Blood, serum, swab, and tissue samples were extracted with the NucleoMag® VET kit for Viral RNA/DNA isolation (MACHEREY-NAGEL, Düren, Germany) on a KingFisher® extraction platform (Thermo Fisher Scientific, Waltham, MA, USA) according to the manufacturer’s instructions. Thereafter, nucleic acids were subjected to the OIE recommended ASFV-specific qPCR according to King et al. [54] with slight modifications. All PCRs were performed using a Bio-Rad C1000^TM^ thermal cycler (BIO-RAD, Hercules, CA, USA), with the CFX96^TM^ Real-Time System of the same manufacturer. Results of qPCR were initially recorded as quantification cycle (cq) values. Using a dilution series of a full-virus ASFV DNA standard, the genome copies in the respective samples were estimated. For generation of the ASFV standard, DNA from an ASFV “Armenia08” macrophage culture supernatant was extracted using the QIAamp Viral RNA Mini Kit (Qiagen, Hilden, Germany) according to the manufacturer’s recommendations. Subsequently, the DNA concentration was determined by spectrophotometry using a Nanodrop 2000c (Thermo Fisher Scientific, Waltham, MA, USA) and the exact number of DNA molecules was calculated with an online tool (http://www.molbiol.edu.ru/eng/scripts/0107.html). Small standard aliquots were stored at −20 °C and thawed not more than five times. The standard was meant to compare the magnitude of viral DNA content rather than giving exact genome copy numbers. 

### 4.4. Detection of ASFV-Specific Antibodies

Serum samples were tested in commercially available ELISAs for the presence of ASFV p72-specific antibodies using the competitive INGEZIM PPA COMPAC ELISA (Ingenasa, Madrid, Spain), for p32-specific antibodies in the ID Screen ASF Competition ELISA (IDVet, Grabels, France), and for antibodies against p32, p62, and p72 using the ID Screen ASF Indirect (IDVet, Grabels, France) Kit according to the manufacturer´s instructions. All serum samples were tested in duplicate. To obtain a value that could be compared to the antibody LFD result using EDTA blood, this matrix was screened in single runs following the protocols provided for serum or plasma.

For confirmatory purposes, all serum and plasma samples were tested in an indirect immunoperoxidase test according to the standard protocols provided by the European Reference Laboratory for ASF with slight modifications regarding the virus strain (standard operating procedure last accessed at SOPs (asf-referencelab.info) on 30 December 2020).

### 4.5. Pen-Side Tests

For pen-side antigen detection, the LFD INgezim ASF CROM Ag (11.ASF.K42, Ingenasa, Madrid, Spain) was used with EDTA blood and serum, following the manufacturer’s instructions. In an attempt to optimize the outcome for EDTA blood samples with negative LFD result but high viral genome load, freeze-thaw cycles and dilution 1:1 in distilled water were attempted for all samples. 

The corresponding LFD INgezim PPA CROM Ab (11.PPA.K.41, Ingenasa, Madrid, Spain) was used on serum and EDTA samples for detection of antibodies against ASFV p72.

### 4.6. Statistical Analysis

Initial data recording and analyses (comparison of mean values, transformation of values) were done using Microsoft Excel 2010 (Microsoft Germany GmbH, Munich, Germany).

GraphPad Prism 8 (Graphpad Software Inc., San Diego, CA, USA) was used for further statistical analyses and graph creation. Statistically significant differences were investigated by paired (for samples taken from the same animal but investigated by different means) or unpaired t-tests (comparison among animals). Statistical significance was defined as *p* < 0.05 and indicated with an asterisk (*), *p* < 0.01 was indicated with two asterisks (**).

## Data Availability

The data that support the findings of this study are available from the corresponding author upon reasonable request.

## Supplementary Materials

The following are available online at https://www.mdpi.com/2076-0817/10/2/177/s1. Figure S1: Detection of ASFV genome by qPCR in blood and organ samples. Figure S2: Comparison of genome copy numbers in different swabs and swab buffers. Figure S3: Comparison of genome copy numbers in alternative sample matrices and standard samples (spleen and EDTA blood). Figure S4: Impressions of the lateral flow devices for the detection of ASFV antigen. Figure S5: Impressions of the lateral flow devices for the detection of ASFV-specific antibodies. Table S1: Overview of the sample set. Table S2: Comparison of antigen LFD and qPCR for detection of ASFV. Table S3: Comparison of antibody LFD with ELISAs and an indirect immunoperoxidase test.
