# Supplementary material for: African Swine Fever Laboratory Diagnosis—Lessons Learned from Recent Animal Trials
Source: Pathogens. 2021 Feb 6;10(2):177. doi: 10.3390/pathogens10020177 (PMC7915929; doi:10.3390/pathogens10020177)
Supplement: Supplementary file 1 [file pathogens-10-00177-s001.zip › Pikalo2021_Supplementary_Figures.pptx]

## Slide 1
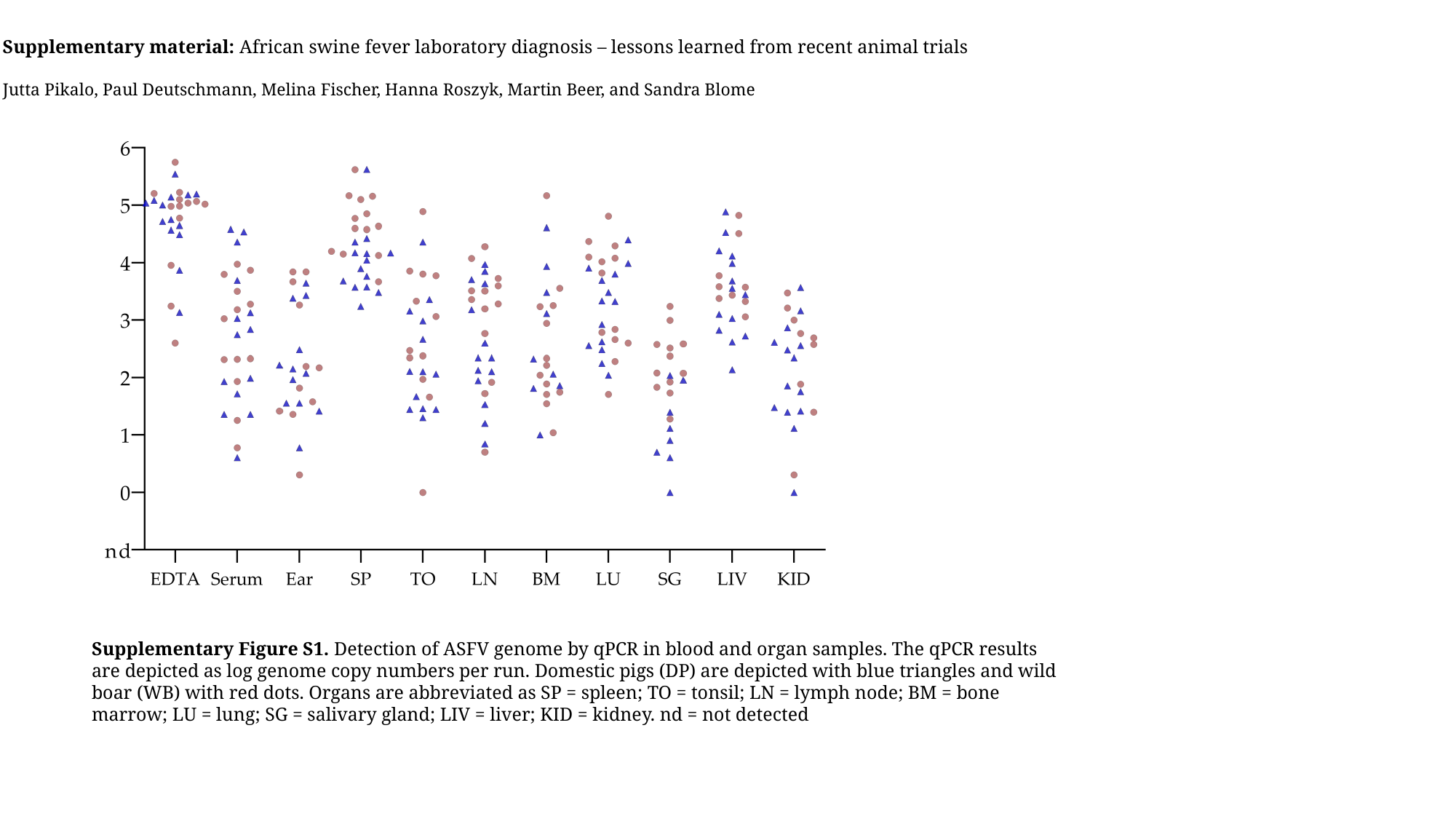

Supplementary material: African swine fever laboratory diagnosis – lessons learned from recent animal trials
Jutta Pikalo, Paul Deutschmann, Melina Fischer, Hanna Roszyk, Martin Beer, and Sandra Blome
Supplementary Figure S1. Detection of ASFV genome by qPCR in blood and organ samples. The qPCR results are depicted as log genome copy numbers per run. Domestic pigs (DP) are depicted with blue triangles and wild boar (WB) with red dots. Organs are abbreviated as SP = spleen; TO = tonsil; LN = lymph node; BM = bone marrow; LU = lung; SG = salivary gland; LIV = liver; KID = kidney. nd = not detected

## Slide 2
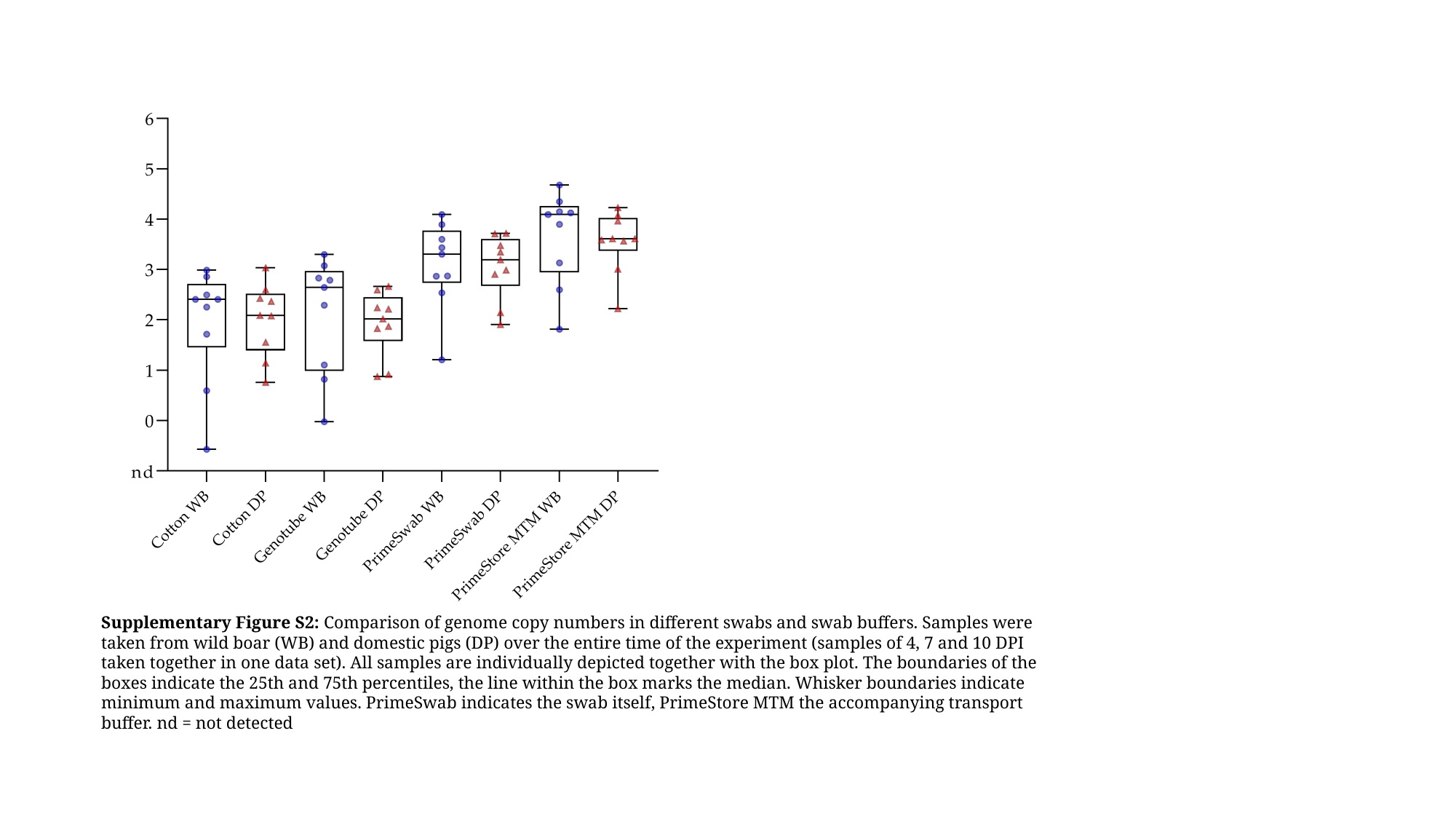

Supplementary Figure S2: Comparison of genome copy numbers in different swabs and swab buffers. Samples were taken from wild boar (WB) and domestic pigs (DP) over the entire time of the experiment (samples of 4, 7 and 10 DPI taken together in one data set). All samples are individually depicted together with the box plot. The boundaries of the boxes indicate the 25th and 75th percentiles, the line within the box marks the median. Whisker boundaries indicate minimum and maximum values. PrimeSwab indicates the swab itself, PrimeStore MTM the accompanying transport buffer. nd = not detected

## Slide 3
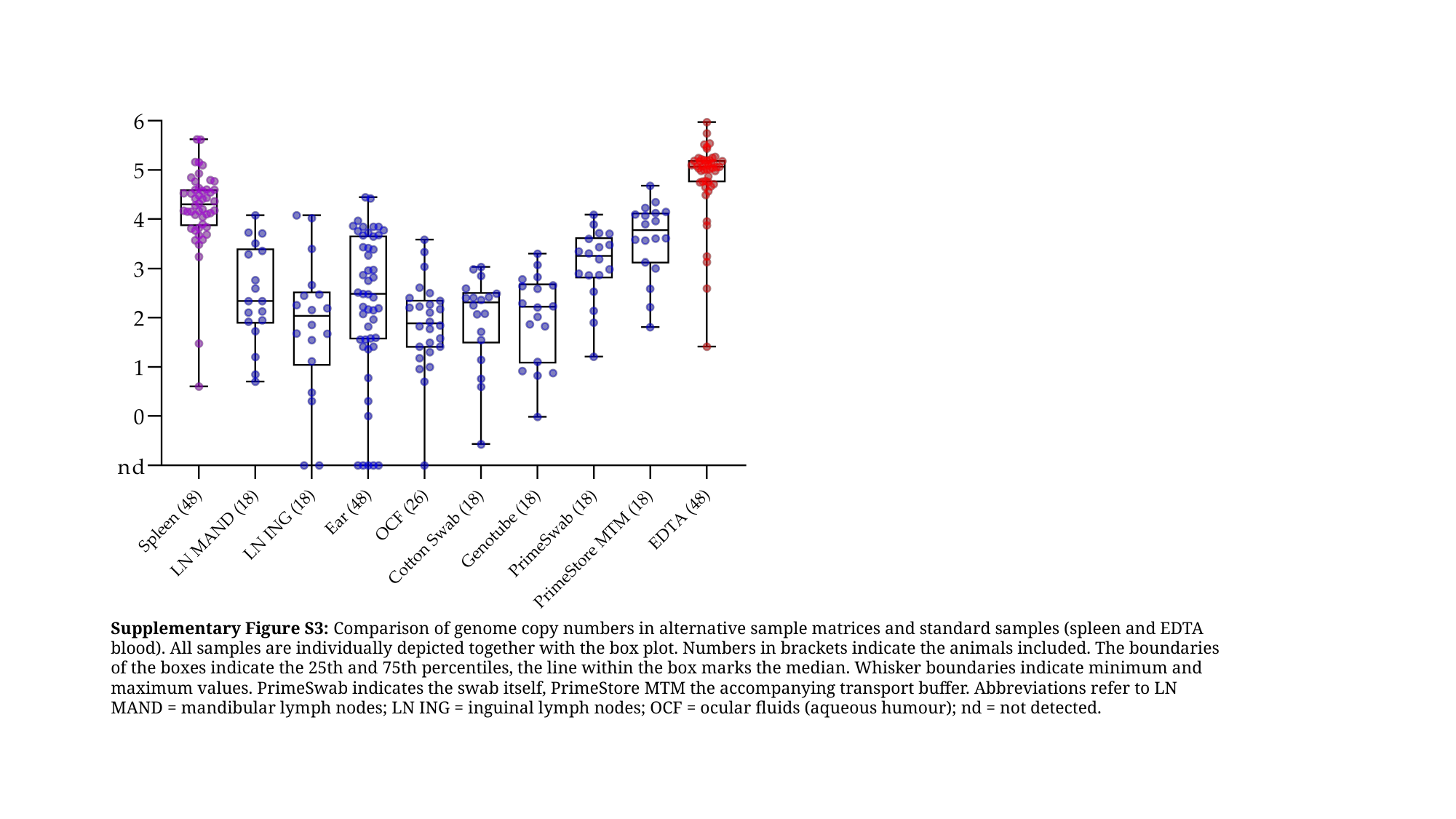

Supplementary Figure S3: Comparison of genome copy numbers in alternative sample matrices and standard samples (spleen and EDTA blood). All samples are individually depicted together with the box plot. Numbers in brackets indicate the animals included. The boundaries of the boxes indicate the 25th and 75th percentiles, the line within the box marks the median. Whisker boundaries indicate minimum and maximum values. PrimeSwab indicates the swab itself, PrimeStore MTM the accompanying transport buffer. Abbreviations refer to LN MAND = mandibular lymph nodes; LN ING = inguinal lymph nodes; OCF = ocular fluids (aqueous humour); nd = not detected.

## Slide 4
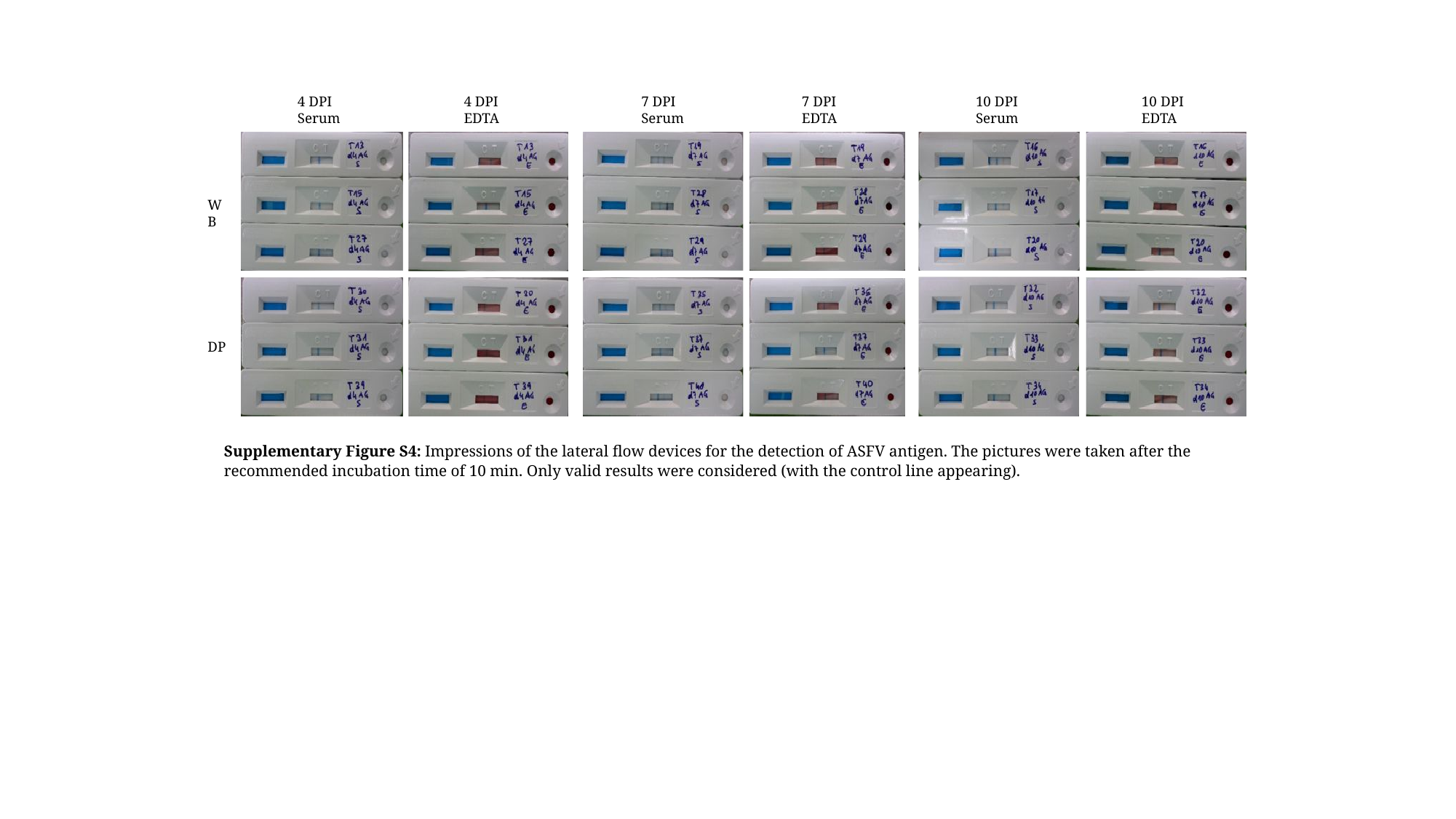

4 DPI
Serum
4 DPI
EDTA
7 DPI
Serum
7 DPI
EDTA
10 DPI
Serum
10 DPI
EDTA
WB
DP
Supplementary Figure S4: Impressions of the lateral flow devices for the detection of ASFV antigen. The pictures were taken after the recommended incubation time of 10 min. Only valid results were considered (with the control line appearing).

## Slide 5
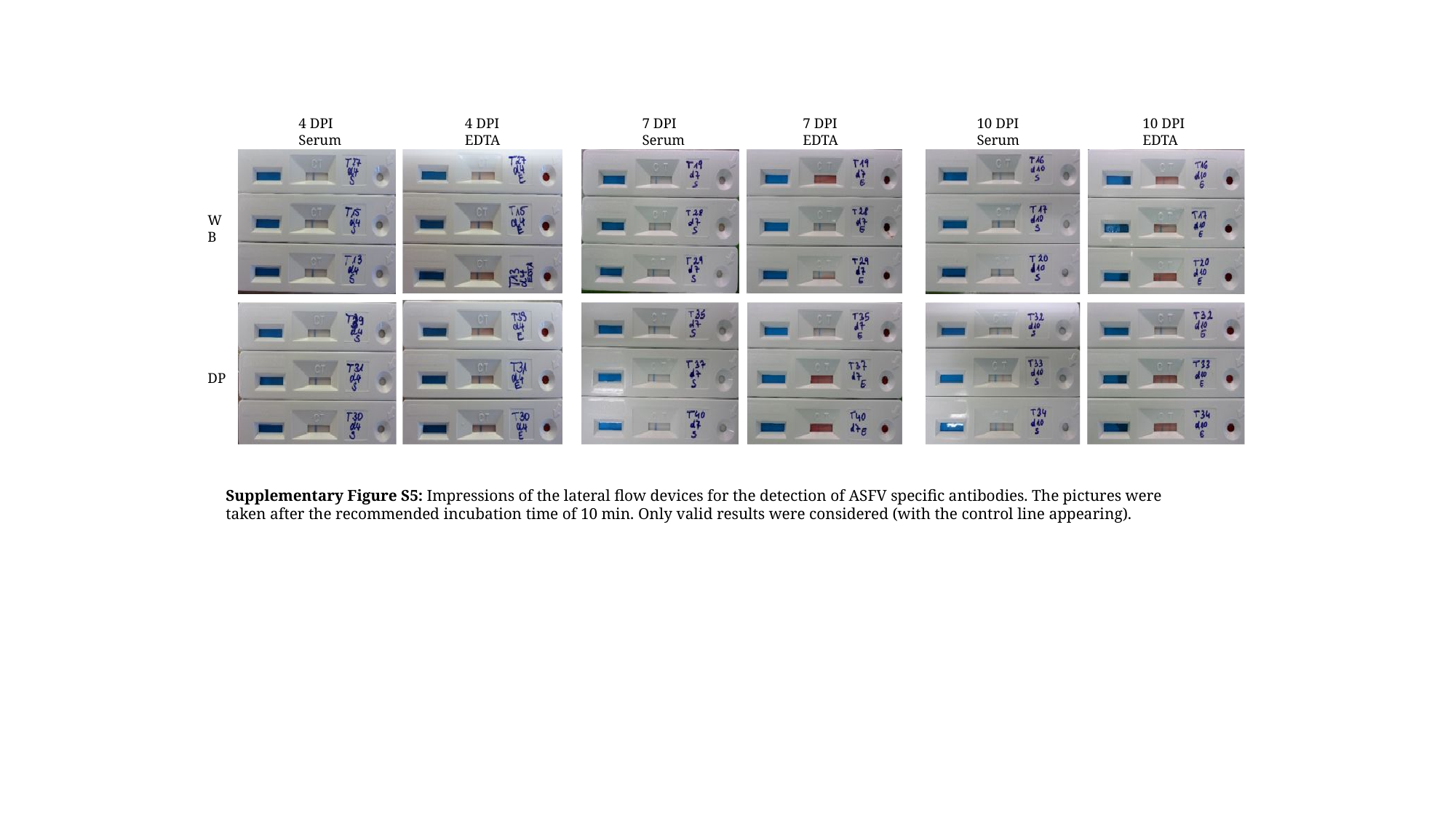

4 DPI
Serum
4 DPI
EDTA
7 DPI
Serum
7 DPI
EDTA
10 DPI
Serum
10 DPI
EDTA
WB
DP
Supplementary Figure S5: Impressions of the lateral flow devices for the detection of ASFV specific antibodies. The pictures were taken after the recommended incubation time of 10 min. Only valid results were considered (with the control line appearing).
